# Evaluation of Abbott RealTime CT/NG Assay for Detection of *Chlamydia trachomatis* and *Neisseria gonorrhoeae* in Cervical Swabs from Female Sex Workers in China

**DOI:** 10.1371/journal.pone.0089658

**Published:** 2014-03-05

**Authors:** Yan Han, Yue-ping Yin, Mei-qin Shi, Bing-jie Zheng, Ming-ying Zhong, Ning Jiang, Shao-chun Chen, Xiang-sheng Chen

**Affiliations:** National Center for STD Control, China Chinese CDC, and Institute of Dermatology, Chinese Academy of Medical Sciences and Peking Union Medical College, Nanjing, China; IPO, Inst Port Oncology, Portugal

## Abstract

**Background:**

To evaluate the performance of the Abbott RealTime CT/NG assay for detection of *Chlamydia trachomatis* (CT) and *Neisseria gonorrhoeae* (NG) among female sex workers (FSWs) in China.

**Methods:**

Cervical swabs from 997 participants were blindly detected by the Abbott RealTime CT/NG assay on the automated m2000 molecular platform and Roche Cobas Amplicor CT/NG assay. Discrepant analysis were confirmed by the Qiagen care CT PCR assay. The sample was defined as candidate nvCT-positive if it was CT positive in the Abbott m2000 assay, but CT negative in the other two assays.

**Results:**

25 specimens that were discordant for CT and 26 specimens that were discordant for NG between the two assays were resolved by Qiagen care CT & NG PCR assays. The sensitivity and specificity, respectively, for Abbott m2000 assay were 92.59% and 100% for CT and 95.45% and 99.90% for NG. The positive predictive value (PPV) and negative predictive value (NPV) of Abbott m2000 assay were100% and 98.52% for CT and 95.5% and 99.90% for NG, respectively. No candidate new-variant CT(nvCT)specimens were identified.

**Conclusion:**

Abbott RealTime CT/NG assay were more specify for CT and NG detection, however, its sensitivity for CT and NG were a little bit lower than Roche Cobas Amplicor CT/NG assay. Abbott RealTime CT/NG assay had higher PPV for NG detection than Roche Cobas Amplicor CT/NG assay; it would be more suitable for screening for population with low-prevalence NG. There is currently no evidence that nvCT is present in FSWs in China.

## Introduction

*Chlamydia trachomatis* (CT) and *Neisseria gonorrhoeae* (NG) are among the most common causes of sexually transmitted infections [1]. Mounting evidence suggests co-infection of CT and NG has been become an urgent phenomenon in many countries. The number of new cases in adults of the CT and NG in 2008 was estimated to be 105.7 million and 106.1 million by the World Health Organization (WHO) [2].

Since 1999 our laboratory has conducted studies to estimate the prevalence of CT and NG infections among high risk groups including FSWs in China using the highly sensitive and specific NAATs-Roche Amplicor PCR assay [3]. This assay is well-established, FDA-cleared method for detection of CT and NG [4]. Despite its advantages, this kit has two major limitations. First, the primers used in its NG analysis, which target the putative cytosine DNA methyltransferase gene, cross react with strains of several commensal Neisseria species. Second, its CT assay, which targets cryptic plasmid, is unable to detect the new variant of CT (nvCT). And this test would be no longer available, and has been replaced with the next generation PCR (Roche Cobas 4800) that detects the CT variant, and has no cross-reactions with other Neisseria. But this Cobas 4800 were not available in China market that time. The FDA-cleared new Abbott RealTime CT/NG assay consists of two different targets for CT detection, targeting both the cryptic plasmid and the chromosomal *omp1* gene. Its NG analysis detects a region in the NG *opacity* (*Opa*) gene. The Abbott RealTime CT/NG assay, which recently became available in China, has been reported to have high sensitivity and specificity, with the advantage of simultaneously detecting both NG and CT (including wild type CT and nvCT) in cervical swab specimens [4]. The nvCT with a 377-base pair (bp) deletion in the cryptic plasmid has been reported in Sweden in 2006 [5].Although the nvCT has been detected in Norway, Finland and Denmark, only a few cases of nvCT have been reported outside the eastern countries [6]. No screening for nvCT have been performed previously in China, despite the fact that the ever-increasing of cross-border travel between China and Sweden.

The report incidence of NG for man and women in American were much higher than that in China in 2011 (98.7 and 108.9 per 100,000 people vs. 17.13 and 14.50 per 100,000 people) [7], [8]. This big difference in report incidence of NG between the United States and China is most likely due to different screening methods, the United States expanded use of more sensitive tests such as nucleic acid amplification testing methods (NAATs) [7] while China routinely based on the less sensitive test such as the Clearview test [9]. As China's National STD Reference Laboratory, we are obligated to recommend the more effective assay as reference test for CT and NG detection. The present study aimed to compare the performances of Abbott RealTime CT/NG assay for the duplex testing of both pathogens from cervical swabs among female sex workers (FSWs) in China. Another goal was to estimate the prevalence of nvCT among this high-risk population.

## Materials and Methods

### Study population

FSWs were recruited from varying work venues in two provinces of southern China (Guangdong and Hainan provinces) from June to September, 2009. A convenience sample of FSWs from each selected venue participated in a questionnaire survey and cervical specimen collection. Females who were older than 18 years and reported providing commercial sex in the past year were eligible for this study. ALL participants provide their written informed consent to participate in this study. The study protocol was reviewed and approved by the ethics committee of Institute of dermatology, Chinese academy of medical sciences and Peking union medical college, Nanjing, China.

### Laboratory test

Two cervical swab specimens were collected by trained nurses from each of the 997 eligible participants. Cervical swab samples were shipped to the National Reference Laboratory of the National Center for STD Control in Nanjing and stored at −70°C until the following analyses. Collection of cervical swabs and preparation of swab eluent were performed according to methods described previously [10]. Of the two swab eluent samples from each participant, one was subjected to the Roche Amplicor CT/NG PCR assay for detection of CT and NG according to the manufacturer's instructions in our laboratory; and the other one was sent to Chinese Abbott consignee's commission laboratory in Beijing, in which it was blind tested with the Abbott RealTime CT/NG assay that uses the automated m2000 molecular platform.

In Roche Amplicor CT/NG PCR system, specimens yielding signals below the negative cutoff for both CT/NG and the internal control (IC) (optical density (OD) <0.2) were interpreted as inhibitory. An inhibitory specimen was retested by processing another aliquot of the original specimen.

The Abbott RealTime CT/NG assay is a qualitative assay. Samples with a cycle number less than or equal to the assay cutoff (CO) were interpreted as “positive.” Samples with a cycle number beyond the assay CO were interpreted as “equivocal.” Samples with no evidence of amplification were interpreted as “negative.” Samples with an initial interpretation of “equivocal” for C. trachomatis were retested.

All samples were tested once initially by both test systems as described above. The samples yielding discrepant results in the two assays were further tested by another technician who had no idea of the primary results using the Qiagen care CT&NG PCR assay. This assay was approved by the State Food and Drug Administration commercially available in China market. Reference standards were set by combining the results of the Roche and Qiagen assays. Samples with an indeterminate CT or NG infection status were considered discrepant results and included in the final assay for evaluation of its sensitivity and specificity.

The Abbott RealTime CT/NG assay was able to detect the nv-CT, while the Roche Amplicor assay and Qiagen care CT assay were unable to detect the nv-CT. The sample was defined as candidate nvCT-positive if it was CT positive in the Abbott m2000 assay, but CT negative in the other two assays. The candidate nvCT-positive will be confirmed by full gene sequence.

### Statistical analysis

Repeatedly inhibitory specimens were excluded from the terminal calculations. All data analyses were performed by IBM SPSS Statistic 20 (SPSS Inc, Chicago, Ill, USA). The sensitivity, specificity, positive predictive value (PPV), and negative predictive value (NPV) of the Abbott assay were calculated using standard methods. The McNemar test was used to compare the results of the Abbott RealTime CT/NG assay with Roche Cobas Amplicor CT/NG assay.

## Results

A total of 997 female cervix swabs were blindly evaluated for CT/NG using the Abbott RealTime CT/NG assay and Roche Cobas Amplicor CT/NG assay.

After inhibited samples were retested using the two assays with the same batch of reagents as that applied initially, 0.8% (8/997) of these specimens gave repetitive inhibitory test results for CT and NG with the Roche Cobas Amplicor CT/NG assay, so these eight specimens were excluded from total CT/NG statistical analysis. Three specimens gave repetitive inhibitory test results for CT with the Abbott RealTime CT/NG assay, so these three specimens were excluded from CT statistical analysis.

Table 1 shows results for 997 cervix swabs tested with both systems for the presence of CT and NG. Using the Roche assay system, 200 specimens were positive for CT (20.2% prevalence) and 46 were positive for NG (4.7% prevalence). Using the Abbott assay, 175 specimens were positive for CT (17.7% prevalence) and 22 were also positive for NG (2.2% prevalence). A total of 13 specimens were positive for both CT and NG using Roche Cobas Amplicor CT/NG assay, 10 were positive for both CT and NG using Abbott RealTime CT/NG assay. Through Roche Cobas Amplicor CT/NG assay, about 28.3% (13.46) NG positive specimens were co-infected with CT, and 6.5% (13/200) CT positive specimens were co-infected with NG. Meantime, when Abbott ReatTime CT/NG assay was used, these rates were 45.5% (10/22) and 5.7% (10/175) respectively.

**Table 1:** Comparison of test results for cervical swabs between Abbott m2000 CT/NG system and Roche Amplicor CT/NG PCR systems.

	Abbott CT					Abbott NG			
Roche CT	Negative	Positive	Indeterminate	Total	Roche NG	Negative	Positive	Indeterminate	Total
Negative	786	0	0	786	Negative	942	1	0	943
Positive	25	175	3	203	Positive	25	21	0	46
Indeterminate	8	0	0	8	Indeterminate	8	0	0	8
Total	819	175	3	997	Total	975	22	0	997

We next calculated concordance of CT between the two assays. There were concordant CT-positive results for 175 specimens and concordant negative results for 786 specimens. 25 specimens were discordant for CT, as they were tested positive with Roche Cobas Amplicor CT/NG assay but negative with the Abbott RealTime CT/NG assay. The positive and negative agreement for CT were 87.5% (95%CI 81.92%, 91.60%) and 100% (95%CI 99.39%, 100%) between the Abbott and Roche assays. The P values from McNemar test for CT was lower than 0.01 between the two systems. The Kappa coefficient for CT was 0.92.

There were concordant NG-positive results for 21 specimens and concordant negative results for 942 specimens. 26 specimens were discordant for NG between the 2 systems: 25 specimens were positive for NG with the Roche Cobas Amplicor CT/NG assay but negative with the Abbott RealTime CT/NG assay; one specimen was negative for NG with Roche Cobas Amplicor CT/NG assay but positive for NG with the Abbott RealTime CT/NG assay. The positive and negative agreement for NG were 45.65% (31.18%, 60.84%) and 99.89% (99.31%, 100%) between the two assays systems. The P values from McNemar test for NG was lower than 0.01. The value of Kappa coefficient for NG was 0.61.

Discrepant analysis of 25 CT Roche positive/Abbott negative specimens; confirmed 12 as true positives by the Qiagen care CT PCR assay. One specimen was confirmed as true positive by the Qiagen care GC PCR assay in discrepant analysis of 25 NG Roche positive/Abbott negative specimens. Discrepant analysis of 1 NG Roche negative/Abbott positive specimens; confirmed 1 as a true negative by the Qiagen care GC PCR assay (Table 2). The overall true positive prevalence of CT and that of NG were 18.97% (187/986, 95% CI, 16.59–21.58%) and 2.22% (22/989, 95% CI, 1.43–3.41%), respectively. The co-infection prevalence of NG and CT were 1.01 (10/986, 95% CI, 0.52–1.92). Three co-infections of NG and CT were missed by the Abbott system and Qiagen system.

**Table 2:** The results of Qiagen care CT&NG PCR assay for discrepant results in Roche and Abbott assays.

	Qiagen CT			Qiagen NG		
	Negative	Positive	Total	Negative	Positive	Total
Roche Pos/Abbott Neg	13	12	25	24	1	25
Roche Neg/Abbott Pos	0	0	0	1	0	1
Total	13	12	25	25	1	26

The performance of Abbott m2000 CT/NG system compared to the reference results (Table 3), the sensitivity and specificity of the Abbott m2000 assay were 92.59% (175/187, 95% CI, 87.63–95.74%) and 100% (799/799, 95% CI, 99.40–100%) for CT and 95.45% (21/22, 95% CI, 75.12–99.76%) and 99.90% (966/967, 95% CI, 99.33–99.99%) for NG, respectively. Its PPV and NPV reached 100% (175/175, 95% CI, 97.32–100%) and 98.52% (799/811, 95% CI, 97.35–99.20%) for CT and 95.5% (21/22, 95% CI, 75.12–99.76%) and 99.90% (966/967, 95% CI, 99.33–99.99%) for NG, respectively. In comparison, The Roche Cobas Amplicor CT/NG assay showed a sensitivity and specificity of 100% (187/187, 95% CI, 97.49–100%) and 98.37% (786/799, 95% CI, 97.16–99.09%) for CT and 100% (22/22, 95% CI, 81.50–100%) and 97.52% (943/967, 95% CI, 96.27–98.37%) for NG, respectively. The PPV and NPV of Roche Cobas Amplicor CT/NG assay were 100% (786/786, 95% CI, 99.39–100%) and 93.50% (187/200, 88.90–96.35%), respectively; for CT and 52.17% (95% CI, 37.12–66.86%) and 100% (943/943, 95% CI, 99.49–100%) for NG.

**Table 3:** The performance of Abbott m2000 CT/NG system compared to the reference results.

	Abbott CT			Abbott NG		
	Negative	Positive	Total	Negative	Positive	Total
Reference Negative	799	0	799	966	1	967
Reference Positive	12	175	187	1	21	22
Total	811	175	986	967	22	989

No candidate nvCT was found among the FSW population in this study.

## Discussion

This is the first independent evaluation study for Abbott RealTime CT/NG assay in China without the support of manufacturers. In the present research, 20.2% and 17.7% specimens were detected CT DNA, 4.7% and 2.2% were detected NG DNA by Roche Cobas Amplicor CT/NG assay and Abbott RealTime CT/NG assay respectively. Given the difference between those assays might be exist, both of them still showed a higher prevalence of CT/NG among the studied population compared with general population [11]–[14]. In addition, a high rate of CT-NG co-infection among NG infected participants partly support the suggestion of the STD Treatment Guideline [15], in which simultaneous treatment to CT among NG-infected patients should be recommended.

The ratio of positive agreement (87.5%) and negative agreement (100%) for CT in this study were comparable to the manufacturer's instruction of the Abbott RealTime CT/NG assay (91.3%, (95% CI, 85.03–95.60%)), (98.9%, (95% CI, 97.38–99.55%).The negative agreement ratio for NG (99.89%) from our study was similar to the manufacturer's instruction of the Abbott RealTime CT/NG assay too. However, the positive agreement ratio for NG was only 45.65% between the Abbott and Roche methods in the population with low prevalence of NG (2.2%), which was significantly lower than the manufacturer's instruction (97.8%, (95%CI, 92.45–99.74%)), and the evaluation study which was supported in part by grant support received from by Abbott Laboratories (96.6% (95%CI, 88.3–99.1%)) among the population with high prevalence of NG (8.2%) [4].

Three co-infections of NG and CT were missed by the Abbott system and Qiagen system. This was probably because the nucleic acid concentration of one target greatly exceeds the other, so PCR reaction failed to amplify the less concentrated target [16]. Alternatively, these three specimens may be false positive. Unfortunately, we could not distinguish between these two possibilities because there was insufficient amount of sample for further analysis.

Abbott RealTime CT/NG assay were more specify for CT and equivalent for NG detection than Roche Cobas Amplicor CT/NG assay. However, Abbott RealTime CT/NG assay sensitivity for CT and NG were some lower than Roche Cobas Amplicor CT/NG assay. Abbott RealTime CT/NG assay had higher PPV for NG detection than Roche Cobas Amplicor CT/NG assay; it would be more suitable for screening for the population with low-prevalence NG. The PPV of Roche Cobas Amplicor CT/NG assay for NG in this study was similar to the study conducted by Diemert 31.6% (13.6–56.5%) in low prevalence (1.19%, 6/503) population [17]. The low PPV in NG of Roche Cobas Amplicor CT/NG assay may be caused by sequence-related issues with the Roche assay. Fortunately, this company has developed the new generation product (Roche 4800) to replace this old assay, the Roche 4800 having a much better performance profile [18], [19].

The nvCT with a 377 bp deletion plasmid has been described in Sweden in 2006. It can cause diseases and has the same biological fitness as wild type CT. It may be able to spread from one country to another in an unnoticed way [20]. However, a range of CT NAAT platforms have been established and utilized in China, which makes the rapid transmission of nvCT unlikely. However, when using the CT NAAT platforms whose primary method for testing CT has no capacity to detect nvCT, epidemiological laboratories need to remain vigilant for obvious decreases in the number of positive CT cases.

Some limitations of this study should be acknowledged. First, the study subjects recruited by convenience sampling were not truly representative of the sex worker population. Second, failure to use Abbott or Roche Standard operation procedures (SOPs) for collection of specimens and storage may affect the performance of these two assays. Third, lack of positive CT variant controls may affect the evaluation of Abbott RealTime CT/NG assay performance for detecting CT variant. Fourth, the Qiagen Care assay is a new assay that has not been widely evaluated and may have low sensitivity, especially for NG detection.

In conclusion, the Abbott RealTime CT/NG assay was comparable for Roche Cobas Amplicor CT/NG assay for CT and NG detection, especially for NG detection in the population with low-prevalence NG. There is currently no evidence that nvCT is present in FSWs in China.

## References

[1] Chen XS, Peeling RW, Yin YP, Mabey DC (2011) The epidemic of sexually trans mitted infections in China: implications for control and future perspectives. BMC Med 9: 111. 21975019 10.1186/1741-7015-9-111 PMC3203037

[2] World Health Organization (2012) Global incidence and prevalence of selected curable sexually transmitted infections - 2008. ISBN 978 92 4 150383 9

[3] Chen XS, Yin YP, Mabey D, Peeling RW, Zhou H, et al (2006) Prevalence of Chlamydia trachomatis infections among women from different settings in China: implications for STD surveillance. Sex Transm Infect 82: 283–284. 16877575 10.1136/sti.2006.019711 PMC2564709

[4] Cheng A, Qian Q, Kirby JE (2011) Evaluation of the Abbott RealTime CT/NG assay in comparison to the Roche Cobas Amplicor CT/NG assay. J Clin Microbiol 49: 1294–1300. 21325546 10.1128/JCM.02595-10 PMC3122843

[5] Ripa T, Nilsson P (2006) A variant of Chlamydia trachomatis with deletion in cryptic plasmid: implications for use of PCR diagnostic tests. Euro Surveill 11: E061109.2. 10.2807/esw.11.45.03076-en 17213548

[6] Unemo M, Clarke IN (2011) The Swedish new variant of Chlamydia trachomatis. Curr Opin Infect Dis 24: 62–69. 21157332 10.1097/QCO.0b013e32834204d5

[7] Centers for Disease Control and Prevention (2012) STD Trends in the United States: 2011.CDC website. Available: http://www.cdc.gov/std/stats11/trends-2011.pdf. Accessed 2014 Feb 8.

[8] National Center for STD Control China CDC (2012) Report of national Syphilis and Gonorrhea epidemiological analysis in 2011. Bulletin for STI Prevention and Control. 258: 9–19.

[9] Yin YP, Peeling RW, Chen XS, Gong KL, Zhou H, et al (2006) Clinic-based evaluation of Clearview Chlamydia MF for detection of Chlamydia trachomatis in vaginal and cervical specimens from women at high risk in China. Sex Transm Infect 82 Suppl 5: v33–v37. 17121763 10.1136/sti.2006.022475 PMC2563916

[10] Gao X, Chen XS, Yin YP, Zhong MY, Shi MQ, et al (2007) Distribution study of Chlamydia trachomatis serovars among high-risk women in China performed using PCR-restriction fragment length polymorphism genotyping. J Clin Microbiol 45: 1185–1189. 17301282 10.1128/JCM.02076-06 PMC1865819

[11] Imai H, Nakao H, Shinohara H, Fujii Y, Tsukino H, et al (2010) Population-based study of asymptomatic infection with Chlamydia trachomatis among female and male students. Int J STD AIDS 21: 362–366. 20498109 10.1258/ijsa.2010.010026

[12] Goulet V, de Barbeyrac B, Raherison S, Prudhomme M, Semaille C, et al (2010) Prevalence of Chlamydia trachomatis: results from the first national population-based survey in France. Sex Transm Infect 86: 263–270. 20660590 10.1136/sti.2009.038752

[13] Torrone EA, Johnson RE, Tian LH, Papp JR, Datta SD, et al (2013) Prevalence of Neisseria gonorrhoeae among persons 14 to 39 years of age, United States, 1999 to 2008. Sex Transm Dis 40: 202–205. 23407466 10.1097/OLQ.0b013e31827c5a71 PMC6893298

[14] Cárcamo CP, Campos PE, García PJ, Hughes JP, Garnett GP, et al (2012) Prevalences of sexually transmitted infections in young adults and female sex workers in Peru: a national population-based survey. Lancet Infect Dis 12: 765–773. 22878023 10.1016/S1473-3099(12)70144-5 PMC3459082

[15] Workowski KA, Berman S, Centers for Disease Control and Prevention (CDC) (2010) Sexually transmitted diseases treatment guidelines, 2010. MMWR Recomm Rep 59: 1–110. 21160459

[16] Marshall R, Chernesky M, Jang D, Hook EW, Cartwright CP, et al (2007) Characteristics of the m2000 automated sample preparation and multiplex real-time PCR system for detection of Chlamydia trachomatis and Neisseria gonorrhoeae. J Clin Microbiol 45: 747–51. 17202273 10.1128/JCM.01956-06 PMC1829145

[17] van Doornum GJ, Schouls LM, Pijl A, Cairo I, Buimer M, et al (2001) Comparison between the LCx Probe system and the COBAS AMPLICOR system for detection of Chlamydia trachomatis and Neisseria gonorrhoeae infections in patients attending a clinic for treatment of sexually transmitted diseases in Amsterdam, The Netherlands. J Clin Microbiol 39: 829–835. 11230391 10.1128/JCM.39.3.829-835.2001 PMC87837

[18] Geelen TH, Rossen JW, Beerens AM, Poort L, Morré SA, et al (2013) Performance of cobas® 4800 and m2000 real-time™ assays for detection of Chlamydia trachomatis and Neisseria gonorrhoeae in rectal and self-collected vaginal specimen. Diagn Microbiol Infect Dis 77: 101–105. 23891224 10.1016/j.diagmicrobio.2013.06.020

[19] Taylor SN, Liesenfeld O, Lillis RA, Body BA, Nye M, et al (2012) Evaluation of the Roche cobas® CT/NG test for detection of Chlamydia trachomatis and Neisseria gonorrhoeae in male urine. Sex Transm Dis 2012 39: 543–549. 10.1097/OLQ.0b013e31824e26ff 22706217

[20] Herrmann B, Törner A, Low N, Klint M, Nilsson A, et al (2008) Emergence and spread of Chlamydia trachomatis variant, Sweden. Emerg Infect Dis 14: 1462–1465. 18760021 10.3201/eid1409.080153 PMC2603114

